# Elucidating Alterations in Viral and Human Gene Expression Due to Human Papillomavirus Integration by Using Multimodal RNA Sequencing

**DOI:** 10.3390/v17101344

**Published:** 2025-10-06

**Authors:** Kana Tamai, Sonoko Kinjo, Ayumi Taguchi, Kazunori Nagasaka, Daisuke Yoshimoto, Anh Quynh Duong, Yoko Yamamoto, Hitoshi Iuchi, Mayuyo Mori, Kenbun Sone, Michiaki Hamada, Kei Kawana, Kazuho Ikeo, Yasushi Hirota, Yutaka Osuga

**Affiliations:** 1 Department of Obstetrics and Gynecology, Graduate School of Medicine, The University of Tokyo, Bunkyo 113-8655, Japan; tamaik-gyn@h.u-tokyo.ac.jp (K.T.);; 2 National Institute of Genetics, Mishima 411-0801, Japan; 3 Laboratory of Human Single Cell Immunology, World Premier International Immunology, Frontier Research Center, Suita 565-0871, Japan; 4 Department of Obstetrics and Gynecology, School of Medicine, Teikyo University, Itabashi 173-8606, Japan; 5 Waseda Research Institute for Science and Engineering, Waseda University, Shinjuku 169-8555, Japan; 6 Computational Bio Big-Data Open Innovation Laboratory (CBBD-OIL), National Institute of Advanced Industrial Science and Technology, Shinjuku 169-8555, Japan; 7 Department of Obstetrics and Gynecology, School of Medicine, Nihon University, Itabashi 173-8610, Japan

**Keywords:** cap analysis of gene expression, human papillomavirus, RNA sequencing, transcription start sites, viral-human chimeric RNA

## Abstract

Human papillomavirus (HPV) infection is a primary driver of cervical cancer. Integration of HPV into the human genome causes persistent expression of viral oncogenes E6 and E7, which promote carcinogenesis and disrupt host genomic function. However, the impact of integration on host gene expression remains incompletely understood. We used multimodal RNA sequencing, combining total RNA-seq and Cap Analysis of Gene Expression (CAGE), to clarify virus–host interactions after HPV integration. HPV-derived transcripts were detected in 17 of 20 clinical samples. In most specimens, transcriptional start sites (TSSs) showed predominant early promoter usage, and transcript patterns differed with detectable E4 RNA region. Notably, the high RNA expressions of E4 region and viral-human chimeric RNAs were mutually exclusive. Chimeric RNAs were identified in 13 of 17 samples, revealing 16 viral integration sites (ISs). CAGE data revealed two patterns of TSS upregulation centered on the ISs: a two-sided pattern (43.8%) and a one-sided pattern (31.3%). Total RNA-seq showed upregulation of 12 putative cancer-related genes near ISs, including *MAGI1-AS1*, *HAS3*, *CASC8*, *BIRC2*, and *MMP12*. These findings indicate that HPV integration drives transcriptional activation near ISs, enhancing expression of adjacent oncogenes. Our study deepens understanding of HPV-induced carcinogenesis and informs precision medicine strategies for cervical cancer.

## 1. Introduction

Cervical cancer is mainly caused by high-risk human papillomavirus (HR-HPV) infection [1,2,3]. In HPV-infected cells, HPV-derived E6 and E7 oncoproteins inactivate p53 and pRb tumor suppressor proteins, which eventually cause resistance to apoptosis and promote cell proliferation [2,4,5]. Continuous expression of E6 and E7 oncoproteins is important for cervical cancer progression [6,7]. HPV integration into the human genome is one of the most influential factors that induces the continuous expression of E6 and E7 oncoproteins.

Traditionally, HPV integration has been studied using techniques such as in situ hybridization and PCR amplification of viral-host fusion sequences or transcripts [8]. More recently, the advent of next-generation sequencing (NGS) technologies—particularly whole genome sequencing and RNA sequencing (RNA-seq)—has enabled more comprehensive identification of integration sites (ISs) [9]. Furthermore, the recent development of NGS has facilitated the gradual elucidation of genetic variations associated with cervical carcinogenesis [10,11]. For example, HPV integration not only induces the continuous expression of E6 and E7 oncoproteins but also triggers various genetic alterations, including oncogenic amplification, chromosomal rearrangements, and chromosomal instability [12,13,14,15]. However, direct evaluation of transcriptional activation surrounding the integration sites has remained challenging.

Cap analysis of gene expression (CAGE) is a transcriptome profiling method that can be used to determine the 5-terminal sequence of RNA, allowing the detection and quantitative measurements of promoter and their activities, respectively [16]. Moreover, CAGE enables the detection of enhancer elements by identifying their transcriptional direction [17,18]. In our previous report on CAGE, we revealed that HPV-derived transcription start sites (TSSs) were altered with cervical cancer progression. TSSs within the early viral promoter became dominant in cervical cancer and high-grade cervical intraepithelial neoplasia (CIN) [19]. CAGE cannot detect the integration of HPV DNA into the human genome or the associated genetic alterations. In contrast, paired-end total RNA-seq can detect transcriptionally active HPV DNA integration sites by identifying viral-human chimeric RNAs [20]. Hence, the combination of CAGE and total RNA-seq may enable us to assess the presence of activated HPV DNA integration sites, the expression of HPV-derived transcriptomes, and TSS upregulation around HPV DNA integration sites.

This study aimed to perform an integrative analysis involving CAGE and total RNA-seq to reveal the genomic status as well as TSS upregulation around integration sites caused by HPV integration into the human genome.

## 2. Materials and Methods

### 2.1. Patients and Clinical Samples

This study was conducted in accordance with the principles of the Declaration of Helsinki. HPV-infected cervical cancer tissue samples were obtained from biopsy or surgical samples. The diagnosis was confirmed by experienced pathologists based on a pathological examination performed at the University of Tokyo Hospital. All experimental procedures were approved by the Institutional Review Board of the University of Tokyo (approval number: G0637), and formal informed consent was obtained from each patient for the use of clinical samples.

### 2.2. HPV Genotyping

DNA was extracted from cervical cancer tissues using the QIAamp DNA Mini Kit (QIAGEN, Hilden, Germany) according to the manufacturer’s instructions. HPV genotyping assays were performed using multiplex polymerase chain reaction (PCR), which is a rapid, high-throughput genotyping procedure that allows the simultaneous detection of 16 types of genital HPV [21], or PCR with PGMY primers followed by reverse line blot hybridization, which allows the detection of 31 types of HPV [22].

### 2.3. Single-Strand CAGE and Total RNA-Seq

Cervical cancer tissues were homogenized in a gentleMACS M Tube (Miltenyi Biotec, Bergisch Gladbach, Germany) with gentleMACS Dissociators (Miltenyi Biotec). Total RNA was isolated using the miRNeasy Mini Kit (QIAGEN). The RNA-seq library was generated using the TruSeq Stranded Total RNA Library Prep kit with Ribo-Zero Human/Mouse/Rat (Illumina, San Diego, CA, USA) using 0.5 μg of total RNA, according to the manufacturer’s protocol. The CAGE library was prepared using 4 μg of total RNA, as previously reported [23]. The cDNA libraries were sequenced using a 50-base paired-end sequence for total RNA-seq and a 50-base single-read sequence for CAGE on an Illumina HiSeq 2500 sequencer (Illumina). The sequencings and base call analyses were performed according to the HiSeq 2500 System Guide using the TruSeq SBS kit v3-HS (Illumina). After the sequencings, raw sequence data were processed using CASAVA-1.8.4, version RTA 1.17.20.0.

### 2.4. Mapping, Quantifying, and Visualizing HPV Transcriptomes

The total RNA-seq and CAGE-seq reads were mapped to the reference genomes of HPV16, HPV18, HPV33, HPV45, HPV52, and HPV58 retrieved from the papillomavirus genome database PaVE (https://pave.niaid.nih.gov/) using TopHat2 v. 2.0.6 [24] and STAR v. 2.7.2b [25], respectively. The mapping results were visualized using IGV.js v2.7.0 [26,27] and implemented on the NGS analysis platform Maser [28]. Based on the mapping results, the expression levels of the reference genomes were estimated according to the PaVE-annotated genes using CuffLinks v 2.0.2 suites [29].

### 2.5. Identification of Viral-Human Chimeric RNA and HPV ISs

Viral-human chimeric RNA was identified as previously reported [20] with slight modifications. Briefly, de novo transcriptome assemblies were performed on each RNA-seq data using Trinity r2013-02-24 [30,31]. The resultant contigs were aligned to the HPV reference genomes using BLASTN (BLAST+ version 2.10.0) with the optional settings of E-value < 1 × 10^−6^ and percent identity ≥98%. Next, the contigs that aligned to the HPV genomes were aligned to the hg38 reference genome (Human G+T database) using the National Center for Biotechnology Information (NCBI) Web BLAST interface according to the criteria described above. Finally, contigs that aligned with the HPV and human reference genomes and whose regions did not overlap were defined as viral-human chimeric RNAs.

In each chimeric RNA, the 5′ end of the region where the chimeric RNA aligned with the hg38 reference genome was considered the IS. Among them, sites at 500 kb or less were bundled, and the 5′ end position in each bundle was defined as the representative HPV IS.

### 2.6. Human Gene Expression Analysis of Total RNA-Seq

The expression levels of human genes were estimated to investigate the transcriptional activity downstream of the IS. Total RNA-seq reads were mapped to the hg38 reference genome using TopHat2 v 2.0.6 [24], and expression levels were calculated based on the NCBI reference sequences (RefSeq) using CuffLinks v 2.0.2 suites. Since topologically associating domains (TADs) are typically up to 1 Mb in size, we analyzed gene expression levels within 500 kb upstream and downstream of the ISs. The genes at 500 kb or less from the IS were defined as the “IS-neighboring genes.”

### 2.7. Quantification of CAGE Reads at the HPV ISs

To investigate transcriptional upregulation around the ISs, CAGE-seq reads mapped to the hg38 reference genome were analyzed using STAR v.2.7.2b [25]. From the mapping results (BAM files), the number of reads located within 1 kb and 10 kb windows centered on each IS was counted. For each read, the mapping start position (4th field in the BAM file) was compared with the genomic position of the IS. Read counts were obtained separately for the 5′ (upstream) and 3′ (downstream) sides, and further divided into sense and antisense strands, as well as their combined totals. To assess whether an IS was associated with transcriptional upregulation, the CAGE read counts were compared among the 20 cases. The case harboring the IS was judged to show upregulation if it had the highest read counts and more than twice the number of counts compared to the 75th percentile of the cohort.

## 3. Results

### 3.1. Transcriptome Analysis of HPV-Derived Transcripts Using CAGE and Total RNA-Seq

Twenty HPV-positive cervical cancer specimens were analyzed using CAGE and total RNA-seq. The clinicopathological characteristics and corresponding HPV genotypes are summarized in Table 1. Sequence data were mapped to hg38 and the corresponding HPV references. The mapping rates of the corresponding HPV references are summarized in Table 1 and Figure S1. Of the 20 cervical cancer cases analyzed, five datasets (Cx02_HPV33, Cx05_HPV52, Cx09_HPV16, Cx15_HPV16, and Cx20_HPV45) had mapping rates to the HPV genome below 0.001%. For Cx09 and Cx15—both small tumor samples—macroscopic sampling for RNA extraction (to preserve tissue for pathology) may have introduced sampling error and led to low HPV detection. The absence of HPV-derived reads in Cx20 remains unexplained, though sampling error cannot be ruled out. Cx02 (HPV33) and Cx05 (HPV52) showed low expression of type-specific gene products but strong HPV16 transcript levels, indicating HPV16 as the primary oncogenic driver. Because our aim was to analyze HPV-derived gene expression, we excluded Cx09, Cx15, and Cx20 from downstream analyses. Detailed sequencing metrics, including total reads, uniquely mapped reads, overall mapping rate, and RNA integrity number, are provided in Table S1.

**Figure 1:**
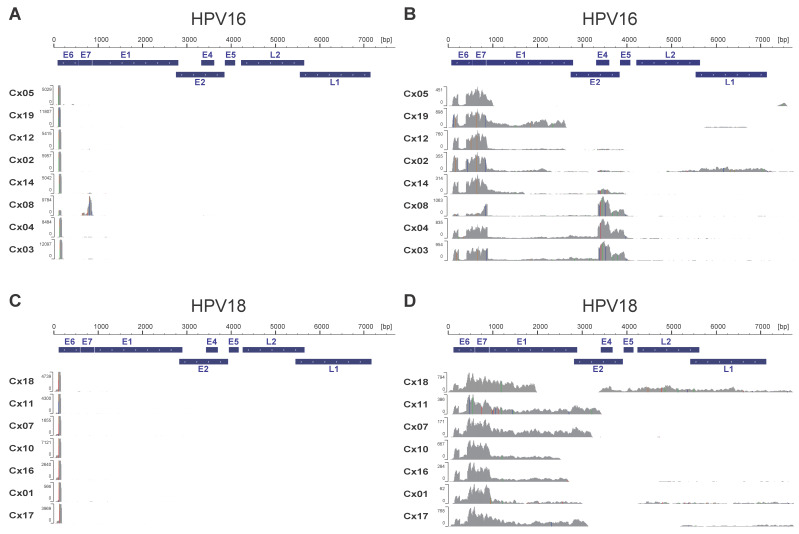
Expression of the HPV-derived transcriptome. CAGE, cap analysis gene expression. CAGE sequence data (**A**) and RNA sequence data (**B**) were mapped to the HPV16 genome and CAGE sequence data (**C**), and RNA sequence data (**D**) were mapped to the HPV18 genome. Mapped data were visualized using IGV.

We visualized HPV-derived TSS activities at the single-nucleotide level. As described in our previous paper, HPV-derived CAGE TSS patterns can be classified into two types: the early promoter-dominant type (Type A), which signifies activated expression of oncogenes E6 and E7, and the late promoter-dominant type (Type B), characterized by the expression of late genes such as L1 and L2, often detected in less severe cases of CIN [19]. Except for the HPV16-positive case (Cx08), all cases were of the prominent early promoter sequence type (Figure 1A,C). Similarly, HPV-derived transcription patterns identified using total RNA-seq were classified into two types according to the expression pattern of detectable E4 RNA regions: an E6/E7 dominant pattern (Type I) and a pattern with the strong expression of E4 RNA regions (Type II). Among HPV16-positive cases, five exhibited the E6/E7 dominant pattern, whereas three showed a strong expression of detectable E4 RNA regions (Figure 1B). In contrast, all HPV18-positive cases showed E6/E7 dominant total RNA-seq patterns, and the expression of E4 RNA regions was reduced (Figure 1D).

### 3.2. Identification of Chimeric RNA

To investigate HPV DNA integration, we detected viral-human chimeric RNAs using an assembly-based approach [20]. A total of 93 chimeric RNAs were identified from 13 samples, including five of eight HPV16-positive cases (63%), all seven HPV18-positive cases (100%), and one HPV52-positive case (Table S2). CAGE patterns, total RNA-seq patterns, and the presence of chimeric RNA in HPV16 and HPV18 cases are summarized in Table 2. All five HPV16-positive cases with detectable chimeric RNAs exhibited a dominant E6/E7 expression pattern in RNA-seq, whereas the three HPV16-positive cases without chimeric RNA detection showed the strong RNA expression of the E4 region. Six of the seven HPV18-positive cases lacked the detectable E4 and E5 RNA regions, suggesting that HPV integration may have occurred upstream of these loci. Conversely, three of the five HPV16-positive cases with chimeric RNAs retained the detectable E4 and E5 RNA regions, indicating that integration did not occur upstream of these regions, or that both integrated and episomal HPV genomes coexist. According to recent findings [32], most splicing variants derived from either the early or late promoters terminate at the early polyadenylation site (pAE) located around position 4215, and these transcripts typically include the E4 and E5 regions. Therefore, the absence of detectable E4 and E5 RNA regions may suggest a disruption or loss of the pAE site. This is also consistent with our observation that detectable E4 RNA region and the presence of chimeric RNAs were mutually exclusive in HPV16-positive cases, suggesting that transcription termination via the viral pAE site may not be functioning properly in the presence of viral integration.

**Figure 2:**
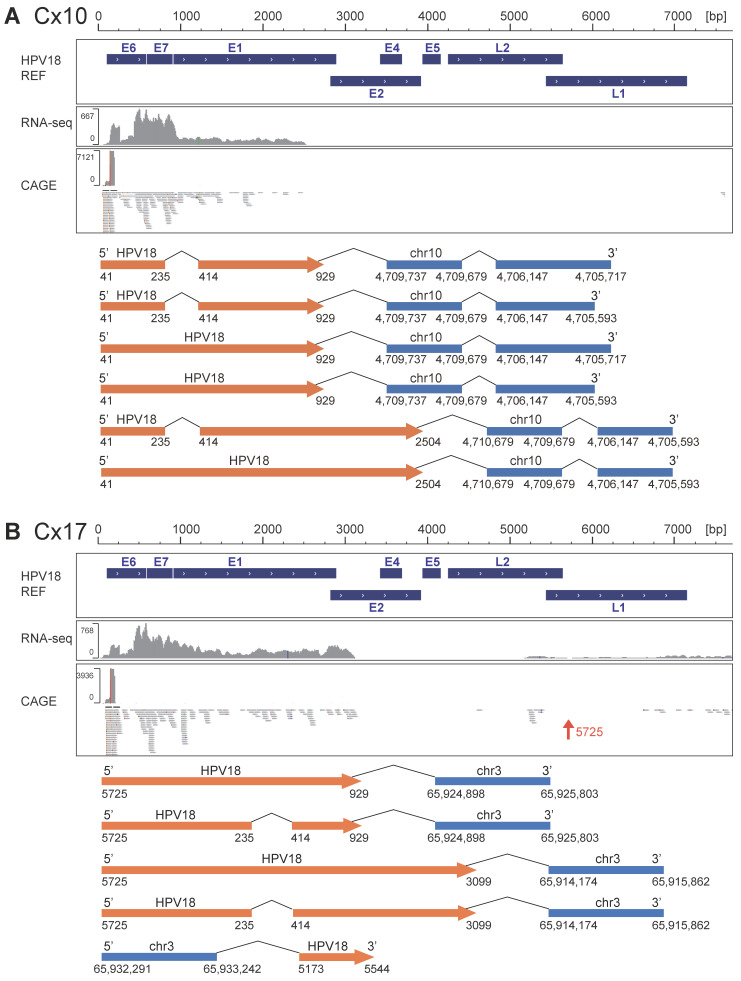
Structures of chimeric RNA. Examples of the chimeric RNA identified using the assembly method are shown. (**A**) Chimeric RNAs starting from the early promoter, (**B**) Chimeric RNAs originating from a specific region of the HPV genome other than the early promoter. Arrows shown in orange indicate the inserted HPV genome direction. RNA-seq and CAGE-mapped data were visualized using IGV.

Subsequently, we focused on the structures of 93 chimeric RNAs. Among them, host-derived RNA was located upstream of HPV-derived RNA in 10 chimeric RNAs, whereas the other 83 chimeric RNAs started from the HPV TSS (Table 2 and Table S2). Among 83 chimeric RNAs with an HPV sequence upstream of the human sequence, 72 started from the long control region of the HPV early promoter. A representative case (Cx10) is shown in Figure 2A. In this case, six chimeric RNAs starting from the HPV early promoter were identified, and CAGE peaks corresponding to the HPV early promoter were identified. However, the remaining 11 chimeric RNAs began from other starting sites (one from nt1377 in Cx02, one from nt6033 in Cx19, four from nt5725 in Cx17, and five from nt6613 in Cx18). Although these 11 chimeric RNAs start from four specific sites on the HPV genome, no corresponding CAGE peaks were observed (Figure 2B and Figure S2), suggesting that these chimeric RNAs may lack 5′ cap structures. However, because CAGE may fail to capture a fraction of capped transcripts due to technical limitations such as library preparation efficiency or RNA structural features, the absence of CAGE peaks does not definitively prove that these RNAs were originally uncapped.

### 3.3. Transcriptome Upregulation of the Chimeric Sites

**Figure 3:**
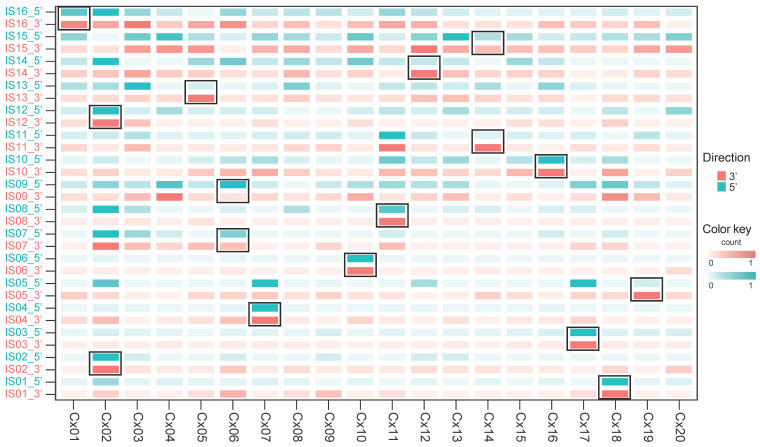
Upregulation of human transcription start sites at HPV integration sites. A heatmap summarizing the upregulation of human transcription start sites (TSSs) located within 10 kb regions upstream (5′, green) and downstream (3′, red) of HPV integration sites (ISs). For each direction, the total number of TSSs was calculated, and values were scaled relative to the sample with the highest count in that direction (set as 1). Color intensity corresponds to the relative TSS activation level. Separate color scales are shown for upstream (green) and downstream (red) regions. HPV-ISs bordered by a thick black square indicate the presence of HPV integration in the corresponding cases.

Some integration sites have been reported to acquire super-enhancer-like structures [33]. We subsequently investigated the upregulation of human TSSs at the ISs and the expression levels of their neighboring genes. For this analysis, ISs located within 500 kb of each other were bundled into one IS, and 16 ISs were identified in 13 samples. First, we compared CAGE read counts within 1 kb and 10 kb windows from each integration site. In the 1 kb window, almost no reads were detected (median = 0, IQR = 0–1, Figure S3A), and therefore this window was excluded from further analyses. In contrast, in the 10 kb window, increased CAGE signals were consistently observed. To investigate the upregulation of human TSSs at the ISs, CAGE reads within a 10 kb window centered on each IS were counted for both 5′ and 3′ sides, and compared within the cohort (Figure 3 and Figure S3B). When analyzed separately on the 5′ and 3′ sides, as well as by strand orientation, the increase in CAGE reads was not restricted to a single strand but detected on both strands (Figure S3B). We found that seven of the 16 ISs (IS_01, IS_02, IS_03, IS_04, IS_06, IS_10, and IS_12) exhibited a two-sided pattern of TSS upregulation, with local upregulation of CAGE reads on both sides of the IS (Figure 3). On the other hand, five ISs showed a one-sided pattern of TSS upregulation (IS_05, IS_08, IS_11, IS_13, and IS_14), with increased CAGE read counts observed only on the downstream (3′) side in all cases. The remaining four ISs did not show TSS upregulation associated with HPV integration (IS_07, IS_09, IS_15, and IS_16).

**Figure 4:**
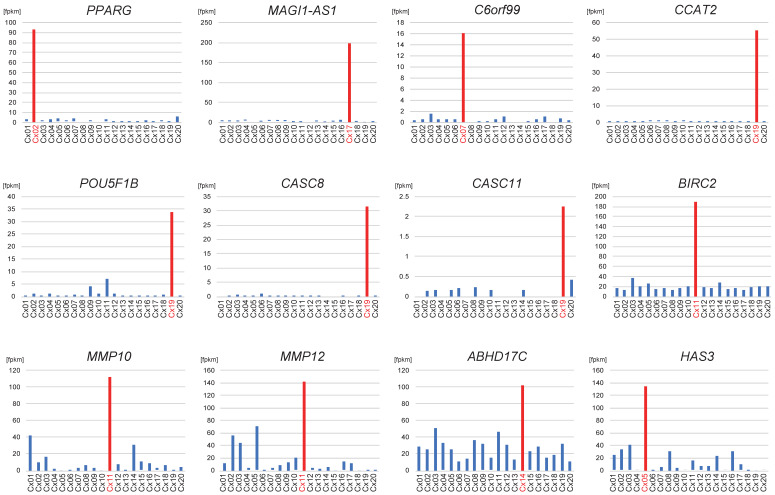
Activation of cancer-related genes around HPV integration sites. Comparison of gene-expression levels around HPV integration sites (ISs) among samples. Cases with the corresponding HPV-IS are marked in red. The 12 genes with increased expression in the cases where each HPV-IS was identified are highlighted.

Neighboring genes were defined as those located within 500 kb of the detected ISs. If the expression level of the target genes in the sample with integration at the corresponding region was more than twice that of the other samples, the gene was identified as being upregulated upon HPV integration. The expression levels of the neighboring genes at each IS are summarized in Table S3. Among the 12 activated ISs, seven led to the upregulation of at least one neighboring gene (Table S3), including PPARG for IS_02 in Cx02, *MAGI1-AS1* for IS_03 in Cx17, *C6orf99* for IS_04 in Cx07, *CCAT2, POU5F1B, CASC8,* and *CASC11* for IS_05 in Cx19, *BIRC2, MMP10*, and *MMP12* for IS_08 in Cx11, *ABHD17C* for IS_11 in Cx14, and *HAS3* for IS_13 in Cx05 (Table S3 and Figure 4). All upregulated genes around the ISs were possibly cancer-related. While HPV frequently integrates into transcriptionally active regions, some TSSs—including those of *PPARG*, *MAGI1-AS1*, *C6orf99*, *CCAT2*, *POU5F1B*, and *CASC8*—were upregulated exclusively in samples with integration at that locus, supporting the possibility that integration contributed to transcriptional activation in these cases.

Among the 17 tumors analyzed, Cx02, Cx14, and Cx06 each harbored two distinct HPV ISs of the same genotype. The transcriptional activity of integrated HPV varies depending on the integration site, and it is known that clones harboring transcriptionally active integration sites preferentially expand [34]. Furthermore, previous studies have suggested that, in tumors with multiple integration events, only one integration site is typically transcriptionally dominant, while the others may be transcriptionally silent [34]. Among the three cases with two ISs, two cases—Cx02 and Cx14—exhibited differences in IS activity. In Cx02 with IS02 and IS12, only the *PPARG* located near IS02 was transcriptionally activated. Similarly, in Cx14 with IS11 and IS15, only *ABHD17C*, located near IS11, was activated, suggesting that IS02 and IS11 are the dominant ISs in Cx02 and Cx14, respectively.

## 4. Discussion

In our study, we conducted multimodal RNA-seq analysis (CAGE and total RNA-seq) and elucidated the transcriptional alteration induced by HPV integration. Especially, we identified two types of TSS upregulation: one in which CAGE read counts increased on both sides of the integration site (two-sided pattern), and another in which increased counts were observed on only one side (one-sided pattern). We also confirmed the enhanced expression of cancer-related genes around ISs.

Consistent with the findings of our previous study, we confirmed that in almost all cancer specimens, HPV-derived TSS exhibited an early promoter-prominent type. Moreover, the patterns of HPV-derived transcripts analyzed using total RNA-seq were classified based on the RNA expression of the E4 region into two types (type I and II). Integration frequently occurs within the E1/E2 region of the HPV genome [35,36,37], and subsequent loss of downstream viral gene expression often leads to the disappearance of E4 RNA region. In this study, we showed that a high expression of the E4 region was mutually exclusive of the presence of viral-human chimeric RNAs. This inverse correlation between the RNA expression of the E4 region and the presence of viral-human chimeric RNAs may be associated with episomal loss in cells with HPV integration [38].

A combination of total RNA-seq and CAGE revealed that although the HPV early promoter was the most activated promoter in almost all cervical cancer samples, regardless of the HPV type, the RNA expression of the E4 region and the presence of chimeric RNA differed between HPV16 and HPV18. In HPV16-positive cases, three of eight cases were negative for chimeric RNA and positive for the RNA expression of the E4 region. Even among cases with HPV integration, the expression of the E4 region was slightly detected in 60% of the cases. In contrast, all HPV18-positive cases were positive for chimeric RNAs and almost negative for the RNA expression of the E4 region. A previous study demonstrated that the viral genome integration rate is higher in HPV18-positive cells than in HPV16-positive ones [39]. Additionally, we had previously demonstrated a low detection rate of E1^E4 RNA region in HPV18-positive cervical precancerous lesions [40], suggesting that HPV18 integration occurs at an early stage of cervical carcinogenesis. Our results reflect the different genomic statuses of HPV16 and HPV18 in cervical cancer cells; specifically, HPV genome integration is observed at a higher frequency in HPV18 than in HPV16.

Some integration sites have been reported to acquire super-enhancer-like structures [33]. In a previous study, a super-enhancer-like region was identified based on the accumulation of BRD4 and H3K27ac. While it is difficult to define super-enhancers solely by CAGE analysis, several studies have reported that active enhancers can be predicted by their characteristic bidirectional TSS activity [17,18]. In our study, TSS upregulation on both sides of the IS, potentially reflecting enhancer-like activity, was observed in seven of 16 ISs. In contrast, a one-sided pattern of TSS upregulation was observed in five ISs, whereas no TSS upregulation was observed in the remaining four ISs. These findings suggest that HPV integration does not necessarily form structures resembling active enhancers, and its effects may vary depending on the integration site and case. Notably, a one-sided pattern of TSS upregulation was also associated with the upregulation of nearby genes via HPV integration. These results indicate that although some integration sites do not generate active enhancers, HPV integration can upregulate nearby cancer-related genes. Therefore, focusing on such integration sites may help elucidate tumorigenic mechanisms and identify novel therapeutic targets.

By combining total RNA-seq and CAGE, we identified 12 HPV integration-associated upregulated genes: *PPARG*, *MAGI1-AS1*, *C6orf99*, *CCAT2*, *POU5F1B*, *CASC8*, *CASC11*, *BIRC2*, *MMP10*, *MMP12*, *ABHD17C*, and *HAS3*. Consistent with previous reports that HPV integration perturbs host gene expression within the same topologically associating domain (TAD) [41], most activated genes mapped directly to IS or lay immediately adjacent, forming continuous regions of transcriptional upregulation. These patterns support the notion that TAD architecture constrains HPV-mediated transcriptional activation. Three genes, *POU5F1B* [11], *MMP12* [42], and *CASC8* [43], have been previously reported as HPV integration targets. The nine novel candidates include *PPARG*, a nuclear receptor involved in differentiation and metabolism and implicated in cancer progression [44,45]; *MMP10*, which promotes tumor invasion via extracellular matrix degradation [46]; and the long noncoding RNAs *CASC11* and *CCAT2*, both regulators of Wnt/β-catenin and MYC signaling pathways [47,48,49,50]. We also identified *BIRC2,* an apoptosis inhibitor and therapeutic target in various cancers [51]; *MAGI1-AS1*, an antisense RNA to the tumor suppressor *MAGI1* that may promote tumorigenesis by suppressing *MAGI1* function [52]; *C6orf99*, a suggested breast cancer biomarker [53]; *ABHD17C*, which participates in protein palmitoylation affecting membrane-associated oncogenic signaling [54]; and *HAS3,* which synthesizes hyaluronan to shape the tumor microenvironment and enhance cancer cell migration [55]. These observations reinforce the idea that HPV integration preferentially occurs near genes with key roles in cancer biology, some of which may represent novel therapeutic targets.

This study has some limitations. First, in our study, we did not perform statistical analyses due to the small sample size. However, this is the first study to demonstrate TSS upregulation around HPV ISs by combining total RNA-seq and CAGE to simultaneously identify active HPV ISs, upregulated neighboring genes, and TSS upregulation at the ISs. Further research is warranted to confirm the association between TSS upregulation in the human genome and the upregulation of neighboring genes. Second, this study analyzed tumor tissues that were pathologically diagnosed as cancer, but whether the dissected cancer tissues used for RNA extraction contained precancerous lesions was not verified. The CAGE TSS shift is more important in precancerous lesions because it reflects cellular differentiation and the steps involved in carcinogenesis [18]. Further research focusing specifically on precancerous lesions is needed to elucidate the alterations in HPV genome status, TSS upregulation, and cervical cancer development. Third, we did not further verify any identified integration sites or mapped CAGE sites by other methods. Fourth, although our study provided detailed structural information on integrated viral transcripts based on total RNA-seq data, we did not perform RACE analysis. Since 3′ or 5′ RACE is necessary to accurately determine the origin of polyadenylated ends and to identify splicing variants [34], the absence of such experiments represents a limitation in the structural characterization of the integrated transcripts.

## 5. Conclusions

By using multimodal RNA-seq analysis combining total RNA-seq and CAGE, we revealed transcriptional upregulation around HPV integration sites and increased expression of adjacent cancer-related genes. This research is important as it elucidated TSS upregulation at HPV integration sites and showed the genomic and transcriptomic profiles of individual cervical cancer cases. The combination of total RNA-seq and CAGE will not only lead to the elucidation of HPV-induced carcinogenesis in individual patients but also mark a breakthrough in precision medicine for patients with cervical cancer.

## Figures and Tables

**Table 1:** Clinicopathological characteristics and summary of the sequences.

Sample ID	Age	Histology	T Stage	LNM	HPV Type	Mapped HPV Type	Total RNA-Seq Reads Mapped to HPV Genome		CAGE Reads Mapped to HPV Genome	
							Read Number	Mapping Rate (%)	Read Number	Mapping Rate (%)
Cx01	67	SCC	2b	0	18	18	397	0.002	614	0.002
Cx02	37	SCC	2a	0	16(33)	16	2589	0.011	6167	0.018
						33	0	0	0	0
Cx03	58	SCC	2b	1	16	16	7086	0.033	13,206	0.045
Cx04	35	ADS	1b1	0	16	16	6091	0.028	9421	0.031
Cx05	36	SCC	2a2	0	16(52)	16	1645	0.008	5235	0.016
						52	0	0	0	0
Cx06	70	SCC	2a2	1	52	52	2717	0.012	5021	0.015
Cx07	34	ADC	1b2	1	18	18	1626	0.007	1895	0.006
Cx08	32	SCC	1b2	1	16	16	4586	0.021	17,587	0.061
Cx09	35	ADS	1b1	0	16	16	3	0.000	160	0.000
Cx10	50	SCC	2b	0	18	18	3970	0.017	8162	0.027
Cx11	32	ADS	1b2	0	18	18	3232	0.015	4805	0.013
Cx12	48	SCC	2b	0	16	16	2927	0.014	5687	0.021
Cx13	43	SCC	1b1	0	58	58	1445	0.007	2351	0.007
Cx14	31	SCC	2a	1	16	16	1546	0.007	5494	0.017
Cx15	48	ADC	1a1	0	16	16	2	0.000	57	0.000
Cx16	29	SCC	2b	1	18	18	1679	0.008	2997	0.009
Cx17	37	SCC	1b	0	18	18	6584	0.031	4576	0.014
Cx18	62	ADC	2b	1	18	18	10,630	0.052	5641	0.016
Cx19	41	ADC	1b2	1	16	16	6905	0.036	12,314	0.045
Cx20	67	ADC	2b	1	45	45	0	0	0	0

ADC, adenocarcinoma; ADS, adenosquamous carcinoma; LNM, lymph node metastasis; SCC, squamous cell carcinoma; CAGE, cap analysis gene expression; RNA-seq, RNA sequencing; HPV type: Genotyping was performed using DNA samples; The total RNA-seq mapping rate was calculated as the number of RNA-seq reads mapped to the HPV genome divided by the total number of RNA-seq reads; the CAGE mapping rate was calculated as the number of CAGE reads mapped to the HPV genome divided by the total number of CAGE reads. Cells shaded in grey indicate samples excluded from downstream analyses.

**Table 2:** Summary of the RNA seq and CAGE patterns and chimeric RNAs.

Sample ID	BINDS ID	HPV Type	TSS Type	Total RNA-Seq Type	Chimeric RNA	Integration Location	Upregulated Genes Around IS	Number of Types of Chimeric RNAs				IS ID	TSS Activation Pattern Around IS
								Total	Start from Human Genome	Start from HPV Genome			
										Start from HPV Early Promoter	Start from HPV Other Promoters		
**Cx02**	BINDS 025-002	16	A	I	+	chr3: 12,405,935	*PPARG*	9	3	5, nt25	1, nt1377	IS 02	bidirectional
					+	chr16: 68,398,335						IS 12	bidirectional
**Cx05**	BINDS 025-005	16	A	I	+	chr16: 69,114,338	*HAS3*	2	0	2, nt27	0	IS 13	unidirectional
**Cx12**	BINDS 025-016	16	A	I	+	chr18: 63,381,192		4	0	4, nt106	0	IS 14	unidirectional
**Cx14**	BINDS 025-018	16	A	I	+	chr15: 80,711,958	*ABHD17C*	9	3	6, nt99	0	IS 11	unidirectional
					+	chr19: 1,879,919						IS 15	none
**Cx19**	BINDS 025-024	16	A	I	+	chr8: 127,417,949	*CASC8* *CCAT2* *POU5F1B* *CASC11*	8	1	6, nt25	1, nt6033	IS 05	unidirectional
**Cx01**	BINDS 025-001	18	A	I	+	chrX: 46,716,828		2	0	2, nt120	0	IS 16	none
**Cx07**	BINDS 025-008	18	A	I	+	chr6: 158,894,981	*C6orf99*	5	0	5, nt43	0	IS 04	bidirectional
**Cx10**	BINDS 025-012	18	A	I	+	chr10: 4,705,593		6	0	6, nt41	0	IS 06	bidirectional
**Cx11**	BINDS 025-015	18	A	I	+	chr11: 102,862,906	*MMP12* *MMP10* *BIRC2*	26	0	26, nt76	0	IS 08	unidirectional
**Cx16**	BINDS 025-020	18	A	I	+	chr15: 71,524,938		7	1	6, nt59	0	IS 10	bidirectional
**Cx17**	BINDS 025-022	18	A	I	+	chr3: 65,914,174	*MAGI1-AS1*	5	1	0	4, nt5725	IS 03	bidirectional
**Cx18**	BINDS 025-023	18	A	I	+	chr2: 176,845,914		6	1	0	5, nt6613	IS 01	bidirectional
**Cx06**	BINDS 025-007	52	A	II	+	chr11: 4,631,073		4	0	4, nt7726	0	IS 07	none
					+	chr12: 52,897,678						IS 09	none
**Cx03**	BINDS 025-003	16	A	II	−			0					
**Cx04**	BINDS 025-004	16	A	II	−			0					
**Cx08**	BINDS 025-010	16	B	II	−			0					
**Cx13**	BINDS 025-017	58	A	II	−			0					

TSS, transcription start site; RNA-seq, RNA sequencing; IS, integration site; TSS type A, the early promoter dominant type; TSS type B, the late promoter dominant type; RNA-seq type I, E6/E7-dominant expression pattern; RNA-seq type II, strong expression in the E4 region; Chimeric RNA, persistence of HPV-human chimeric RNA. The columns “Start from HPV early promoter” and “Start from HPV other promoters” show the number and start site of chimeric RNAs from each promoter. For example, (5, nt25) indicates five chimeric RNAs starting at nucleotide 25 of the HPV genome.

## Data Availability

The sequence data used in this study has been deposited at the Japanese Genotype-phenotype Archive (JGA, https://www.ddbj.nig.ac.jp/jga (accessed on 25 July 2025)), which is hosted by the Bioinformation and DDBJ Center, under accession number JGAS000822.

## Supplementary Materials

The following supporting information can be downloaded at: https://www.mdpi.com/article/10.3390/v17101344/s1, Table S1: The number of sequenced reads and mapping rate of RNA sequencing; Table S2: Mapping information of chimeric RNA; Table S3: List of integration sites and gene expression around integration sites; Figure S1: Mapping rate against HPV genome; Figure S2: Structure of chimeric RNAs originating from HPV genome other than early promoter regions; Figure S3: (A) CAGE read counts around HPV integration sites in 1 kb and 10 kb, (B) CAGE read counts within 10 kb windows from integration sites by 5′/3′ position and sense/antisense orientation.

## Abbreviations

The following abbreviations are used in this manuscript:

BINDS	Basis for Supporting Innovative Drug Discovery and Life Science
CAGE	cap analysis of gene expression
CIN	Cervical intraepithelial neoplasia
HPV	human papillomavirus
IGV	Integrative Genomics Viewer
IS	Integration Site
NCBI	National Center for Biotechnology Information
NGS	next-generation sequencing
ORF	open reading frame
PCR	polymerase chain reaction
RNA-seq	RNA sequencing
RIN	RNA integrity number
TAD	Topologically associating domain
TSS	transcription start sites
