# Supplementary figures and images for: Elucidating Alterations in Viral and Human Gene Expression Due to Human Papillomavirus Integration by Using Multimodal RNA Sequencing
Source: Viruses. 2025 Oct 6;17(10):1344. doi: 10.3390/v17101344 (PMC12567678; doi:10.3390/v17101344)

Fig. S2

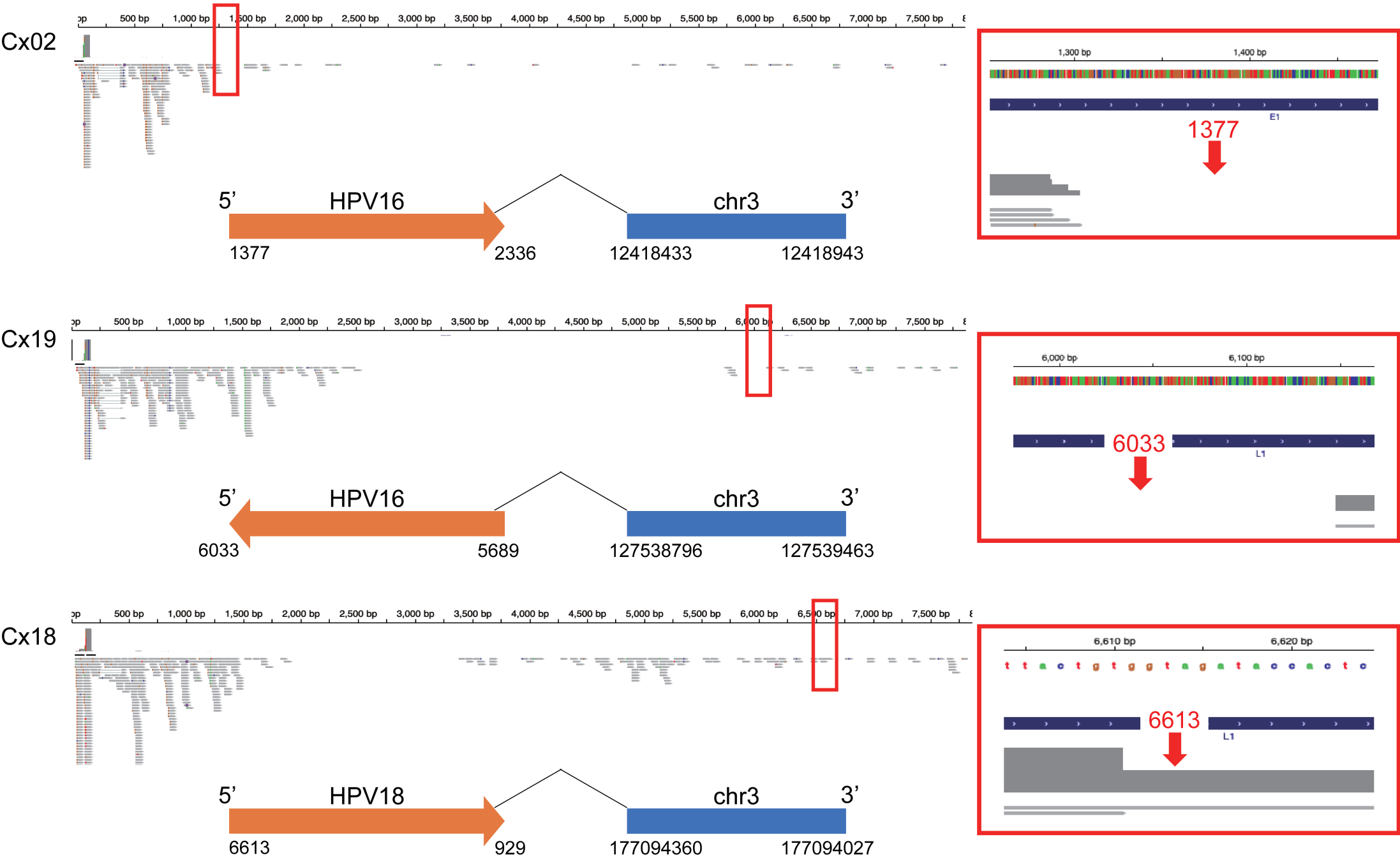

Supplement: Supplementary file 1 [file viruses-17-01344-s001.zip › Supplement/Fig.S2 (600dpi).pdf]

Fig. S1

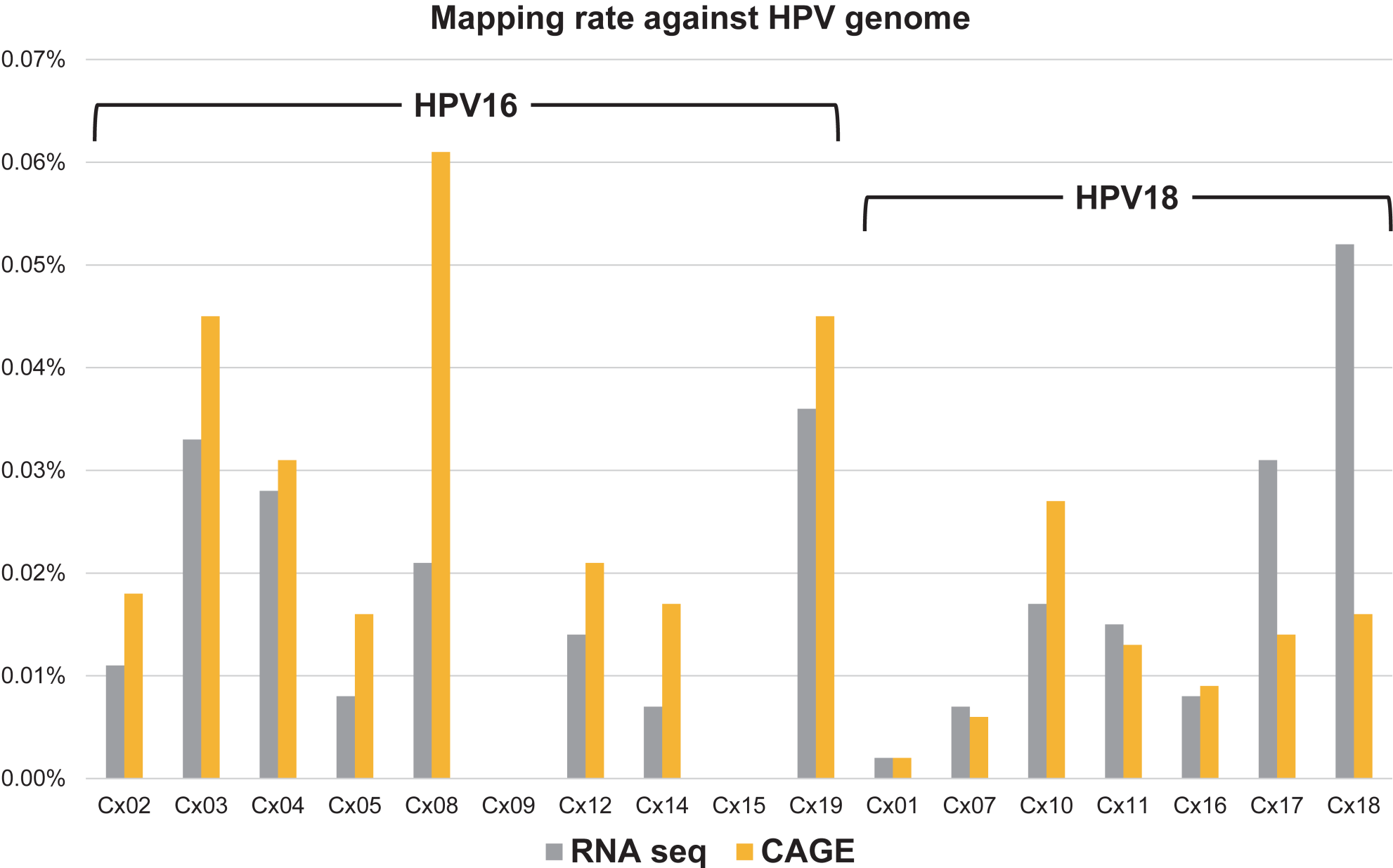

Supplement: Supplementary file 1 [file viruses-17-01344-s001.zip › Supplement/Fig.S1 (600dpi).pdf]

**Fig. S3**

**A**

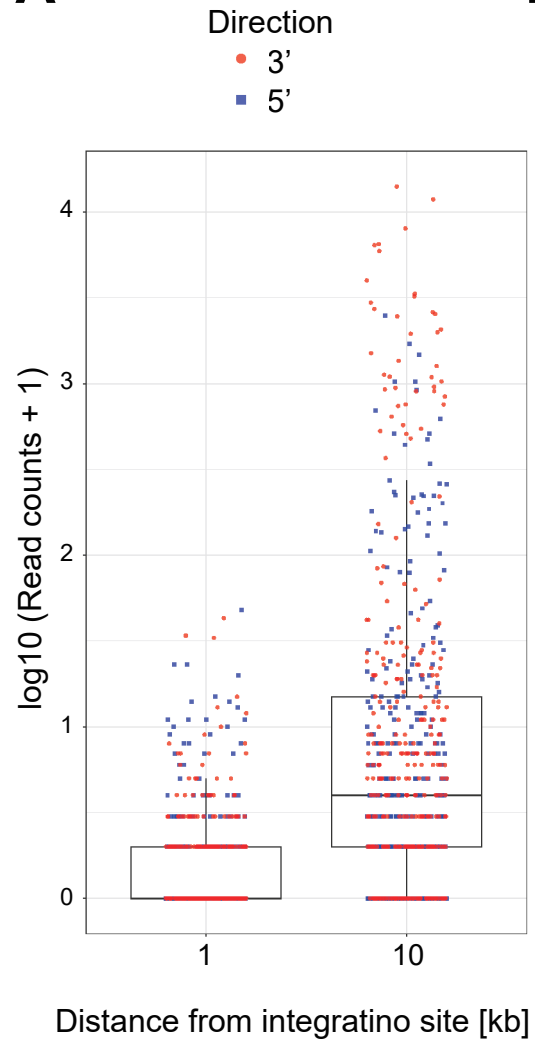

**B**

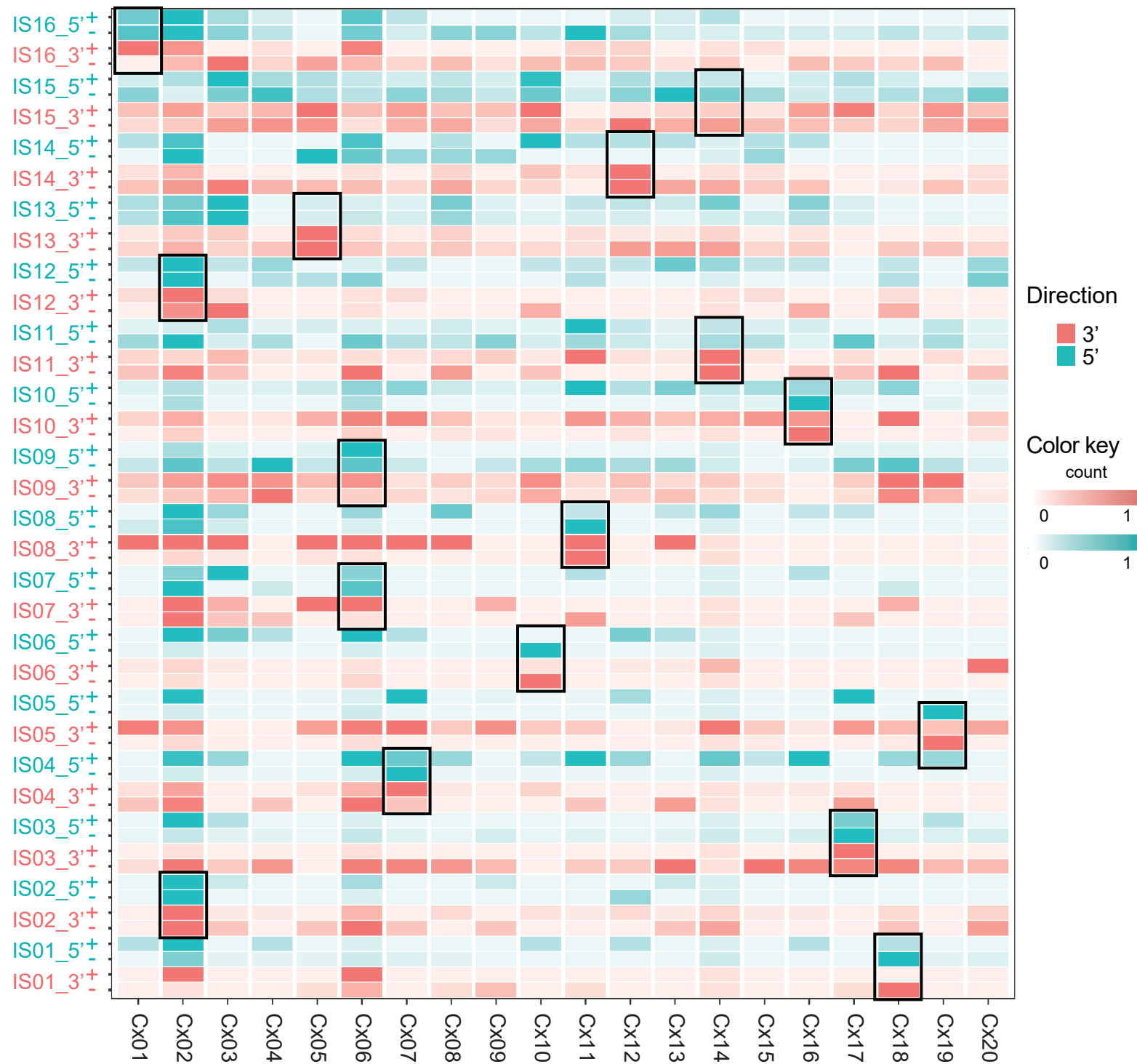

Supplement: Supplementary file 1 [file viruses-17-01344-s001.zip › Supplement/Fig.S3.pdf]
